# Supplementary material for: NHS Diabetes Prevention Programme in England: formative evaluation of the programme in early phase implementation
Source: BMJ Open. 2018 Feb 21;8(2):e019467. doi: 10.1136/bmjopen-2017-019467 (PMC5855311; doi:10.1136/bmjopen-2017-019467)
Supplement: Supplementary file 1 [file bmjopen-2017-019467supp001.pdf]

Components of the NHS DPP intensive lifestyle change intervention as detailed by First wave provider organisations in programme documents: comparison to NICE PH38 guidance and the NHS DPP National Service Specification

Intervention specification	NICE PH38	NHS DPP *	1	2	3	4
**Components**						
Weight Management	✓	✓	✓	✓	✓	✓
Physical activity	✓	✓	✓	✓	✓	✓
Diet	✓	✓	✓	✓	✓	✓
Smoking Cessation	✗	✓	✓	✓	✓	✓
Tailoring	✓	✓	✓	✓	✓	✓
Links to services/integrated approach		✓	✓	✓	✓	✓
**Providers**						
Primary healthcare professionals/ NHS specialist	✓	✓	✗	✗	✗	✓
Health educators	✓	✓	✓	✓	✓	✓
**Setting**						
Range of venues in the community	✓	✓	✓	✓	✓	✓
**Format**						
Group	✓	✓	✓	✓	✓	✓
Up to 20 participants	✓	✓	✓	✗	✗	✓
Face-to-face	✓	✓	✓	✓	✓	✓
Individual	✗	✓	✓	✓	✓	✓
Digital (as supportive addition)	✗	✓	✓	✓	✓	✓
**Type of sessions**						
Counselling (person-centred approach)	✓	✓	✓	✗	✓	✗
Educational	✓	✓	✓	✓	✓	✓
Behaviour change skills training		✓	✓	✓	✓	✓
Experiential (e.g. physical activity)	✗	✓	✓	✓	✓	✓
**Behaviour Change Techniques (BCT)**						
Use of taxonomy of BCTs		✓	✓	✓	✓	✓
Information provision	✓	✓	✓	✓	✓	✓
Goal setting	✓	✓	✓	✓	✓	✓
Self-monitoring		✓	✓	✓	✓	✓
Provision of feedback			✓	✗	✓	✓
Action planning	✓	✗	✓	✓	✓	✓
Coping planning	✓	✗	✓	✗	✓	✗
Problem solving/ Relapse prevention	✓	✗	✓	✓	✓	✓
Goal revision	✓	✓	✓	✓	✓	✓
Social support	✓	✗	✓	✓	✓	✗
**Use of behaviour change theory**						
Identification and rationale		✓	✓	✓	✓	✓
Map to BCTs		✓	✓	✓	✓	✓
Map to behaviours		✓	✓	✓	✓	✓
Framework used to select theories/predictors and BCTs		✓	✗	✗	✓	✓
**Duration**						
Minimum 9-18 months	✓	✓	✓	✓	✗	✓
**Intensity**						
Minimum 13 sessions (minimum 16 hours)	✓	✓	✓	✓	✗	✓
Core sessions - Weekly or fortnightly	✓	✓	✓	✓	✓	✓

✓ Included; ✗ Not reported

* NHS DPP First Wave service specification, 1-4 NHS DPP first wave provider organisations
